# Changes in the mean incidence and variance of orthopedic diseases before and during the COVID-19 pandemic in Korea: a retrospective study

**DOI:** 10.1186/s12891-023-06634-0

**Published:** 2023-07-01

**Authors:** Joo-Hee Kim, Mi Jung Kwon, Hyo Geun Choi, Sang Jun Lee, Sangwon Hwang, Jaemin Lee, San-Hui Lee, Jung Woo Lee

**Affiliations:** 1 Division of Pulmonary, Allergy, and Critical Care Medicine, Department of Medicine, Hallym University College of Medicine, 22, Gwanpyeong-ro 170beon-gil, Dongan-gu, Anyang, Korea; 2 Department of Pathology, Hallym University Sacred Heart Hospital, Hallym University College of Medicine, Anyang, Korea; 3 Suseoseoulent clinic, Seoul, Korea; 4 Yonsei University Wonju College of Medicine, 20, Ilsan-ro, Wonju-si, Gangwon-do Korea; 5 Artificial Intelligence Bigdata Medical Center, Yonsei University Wonju College of Medicine, 20, Ilsan-ro, Wonju-si, Gangwon-do Korea; 6 Department of Orthopedic Surgery, Yonsei University Wonju College of Medicine, 20, Ilsan-ro, Wonju-si, Gangwon-do Korea; 7 Department of Obstetrics and Gynecology, Yonsei University Wonju College of Medicine, 20, Ilsan-ro, Wonju-si, Gangwon-do Korea

**Keywords:** Musculoskeletal diseases, Orthopedic diseases, COVID-19, Gout, Myofascial pain syndromes, Frozen shoulder

## Abstract

**Background:**

During the coronavirus disease (COVID-19) pandemic, the amount of moderate- to high-intensity physical activity significantly decreased. Therefore, the epidemiology of musculoskeletal diseases could possibly have changed. We assessed changes in the incidence of and variance in non-traumatic orthopedic diseases before and after the COVID-19 pandemic in Korea.

**Methods:**

This study included data from the Korea National Health Insurance Service, which covers the entire Korean population (approximately 50 million), from January 2018 to June 2021. Using International Classification of Diseases, Tenth Revision codes, 12 common orthopedic diseases were evaluated, including cervical disc disorders, lumbar disc disorders, forward head posture, myofascial pain syndrome, carpal tunnel syndrome, tennis elbow, frozen shoulder, rheumatoid arthritis, gout, hip fracture, distal radius fracture, and spine fracture diseases. “Pre-COVID-19” was the period until February 2020, and “COVID-19 pandemic period” was the period starting March 2020. Differences in the mean incidence and variance of diseases before and during the COVID-19 pandemic were compared.

**Results:**

In most cases, the incidence of orthopedic diseases decreased at the beginning of the pandemic and then increased thereafter. Among the 12 diseases, the incidence of three diseases showed a statistically significant change. The incidence of myofascial pain syndrome (*P* < 0.001) was lower during the COVID-19 pandemic than during the pre-COVID-19 period. The incidences of frozen shoulder (*P* < 0.001) and gout (*P* = 0.043) were higher during the COVID-19 pandemic than during the pre-COVID-19 period. However, no statistical difference in disease variations was observed between the two periods.

**Conclusions:**

The incidence of orthopedic diseases varied during the COVID-19 pandemic among the Korean population. Although the incidence of myofascial pain syndrome was lower, that of frozen shoulder and gout was higher during the COVID-19 pandemic than during the pre-COVID-19 period. No disease variations during the COVID-19 pandemic were found.

**Supplementary Information:**

The online version contains supplementary material available at 10.1186/s12891-023-06634-0.

## Background

Severe acute respiratory syndrome coronavirus 2, which causes coronavirus disease (COVID-19), first appeared in Wuhan, China, in December 2019 [1] and gradually spread globally. Breakthrough infections have been reported among fully vaccinated healthcare workers [2]. As asymptomatic infections may pose a risk to vulnerable populations, quarantine and lockdown measures were established to control the COVID pandemic. However, social isolation prevented citizens from going out and undertaking their routine activities and consequently harmed the global economic situation [3]. Furthermore, these health restrictions also have a negative impact on human psychology: people experienced symptoms of distress, depression, posttraumatic stress disorder, anxiety, frustration, and suicide [4].

Social restrictions or lockdown included vacation for schools and working from home [3]. Several papers that analyzed a small number of participants have been published in Korea. In the early phase of the pandemic, 96.7% and 83.4% of survey respondents avoided outdoor activities and crowded places, respectively [5]. Restricting people’s activity represents another health problem. The duration of moderate- to high-intensity physical activity significantly decreased (by 4.93–21.18 min) after the outbreak in Korea [6]. Similar trends have been reported in other countries. In a study of 35 research organizations from Europe, North Africa, Western Asia, and the Americas, daily sitting time increased from 5 to 8 h per day [7]. In physically active older Brazilian women, body weight (*P* = 0.002) and body mass index (*P* = 0.001) increased significantly, and muscle function loss increased from 13.8 to 27.6% after 1 year of lockdown [8]. These changes in activity range and amount can alter the epidemiology of musculoskeletal disease.

We hypothesized that the incidence and variance of a wide range of orthopedic diseases might have changed during the COVID-19 era. To test this hypothesis, we compared the number and variations in the incidence of multiple orthopedic diseases before and during the COVID-19 pandemic period in this study. According to a meta-analysis published at the beginning of the pandemic, sports-related traumas were less frequent than those before the pandemic [9]. Although a review paper was published after the meta-analysis, studies on common non-traumatic diseases are rare [10]. We evaluated the monthly incidence of common orthopedic diseases in primary clinics and compared the incidence and seasonal variations between the pre-COVID-19 and COVID-19 pandemic periods. As sex and age may have impact on the disease incidence, we also performed subgroup analyses by sex and age.

## Methods

### Participants and measurement

The entire Korean population (approximately 50 million) is registered in the Korean National Health Insurance Service (NHIS). We used all medical records from NHIS, which include public and private information on the population’s demographics, medical use, and claim database. There were no exclusion criteria because the NHIS covers the whole population (e.g., 51,780,000 people in 2019) and records data of all Koreans from primary clinics to tertiary hospitals. In this study, we selected 12 orthopedic diseases common in primary clinics and evaluated the monthly incidence of each disease from January 2018 to June 2021. The study duration was divided into two periods: “pre-COVID-19” (until February 2020) and “COVID-19 pandemic” (from March 2020). This is because the first patients with COVID-19 were identified on January 20, 2020, and measures for disease prevention and control started on March 2020 in Korea.

All diagnoses were accessed by their International Classification of Diseases, Tenth Revision (ICD-10) codes: cervical disc disorders (M50), lumbar disc disorders (M51), forward head posture (S134), myofascial pain syndrome (M791), carpal tunnel syndrome (G560), tennis elbow (M771), frozen shoulder (M750), rheumatoid arthritis (M05, M06), gout (M10), hip fracture (S720, S721, S722), distal radius fracture (S525), and spine fracture (S220, S320).

As this study included the entire medical records of hospitals or clinics in Korea, there was no duplication of the incidence of disease and patient identification. All patients had their own registration number and were uniquely identified. For the incidence estimates, the date of the earliest claim with a registration code was defined as the index date and was considered the incident time, and the patient was considered an incident case in that year.

### Statistics

Statistical analyses were performed using SPSS version 22.0 (IBM, Armonk, NY, USA). We compared the mean incidence of diseases by month between the two periods (pre-COVID-19 and COVID-19 pandemic periods) using the Mann–Whitney U test for nonparametric values. The difference in variance of diseases between the two periods (standard deviation) was compared using Levene’s test for nonparametric values [11]. Regarding the subgroup analyses, we divided the participants by age (0–19, 20–59, and ≥ 60 years old) and sex. We divided the age groups of the participants into minors before 20 years of age [12], youth and middle-aged groups up to 60 years of age [13], and senior groups over 60 years of age [14, 15], in consultation with the authors. Two-tailed analyses were performed to determine statistical significance, and *P*-values < 0.05 were considered significant.

## Results

### Incidence of Orthopedic Diseases

**Fig. 1:**
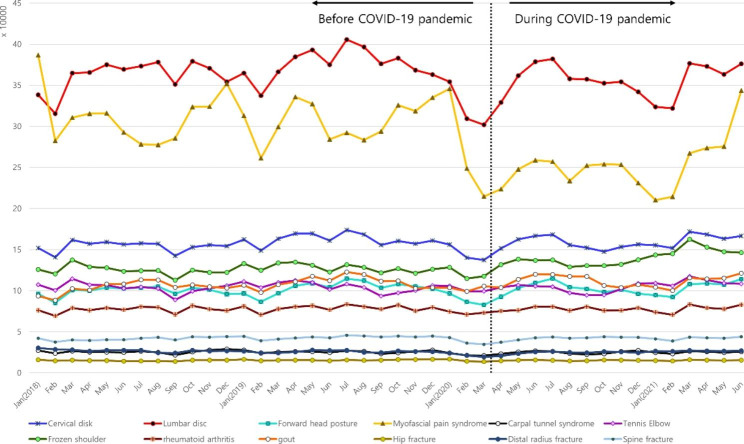
Monthly incidence of orthopedic diseases in 2018, 2019, 2020, and 2021. The study duration was divided into two periods: before the COVID-19 pandemic (until February 2020) and during the COVID-19 pandemic (from March 2020)

**Fig. 2:**
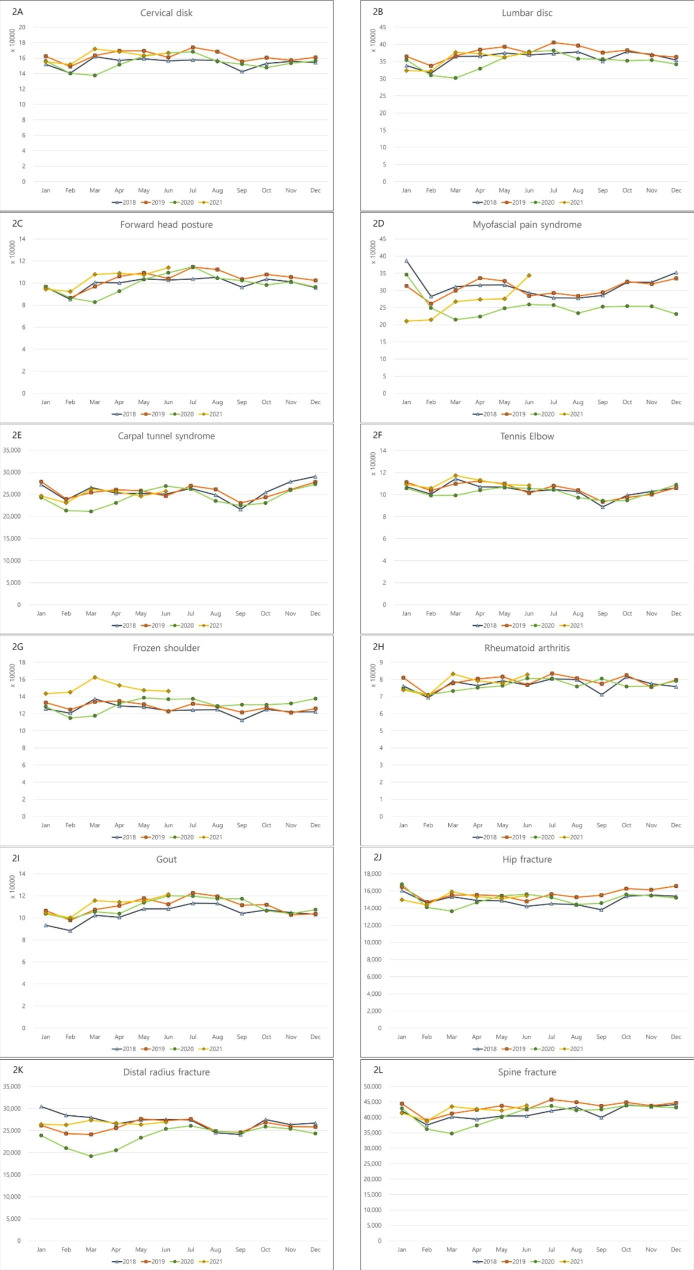
Monthly incidence of orthopedic diseases in 2018, 2019, 2020, and 2021: (**A**) cervical disc disorder, (**B**) lumbar disc disorder, (**C**) forward head posture, (**D**) myofascial pain syndrome, (**E**) carpal tunnel syndrome, (**F**) tennis elbow, (**G**) frozen shoulder, (**H**) rheumatoid arthritis, (**I**) gout, (**J**) hip fracture, (**K**) distal radius fracture, and (**L**) spine fracture

Most diseases did not show seasonality before the COVID-19 pandemic, with a slightly decreased incidence in September and February (Fig. 1). Lumbar disc disorder and myofascial pain syndrome had the highest incidence among the 12 diseases. The incidence of both conditions showed small peaks during summer (July and August) and winter (December and January) and decreased at the beginning of the COVID-19 pandemic (February and March 2020). The incidence of lumbar disc disorder soon increased after several months, and the incidence of myofascial pain syndrome increased starting on February 2021 (Fig. 2). From the first year after the pandemic, eight diseases with the highest incidence showed an increasing incidence pattern. Additional files 1 to 3 present these data in more detail.

Among the 12 diseases, the incidence of three diseases had a statistically significant change. The incidence of myofascial pain syndrome (*P* < 0.001) was lower during the COVID-19 pandemic than during the pre-COVID-19 period. In addition, frozen shoulder (*P* < 0.001) and gout (*P* = 0.043) had higher incidences during the COVID-19 pandemic than during the pre-COVID-19 period (Table 1).

**Table 1:** Incidence of diseases before and during COVID-19 and their difference

Diseases	Before COVID-19		During COVID-19		*P*-values of difference	
	Mean	SD	Mean	SD	Mean	Variance^†^
Cervical disc disorder	157,468.0	8332.4	158,053.8	9256.8	0.917	0.139
Lumbar disc disorder	365,856.3	22,250.6	353,236.8	23,587.9	0.097	0.977
Forward head posture	100,857.5	7288.7	101,865.9	8720.7	0.679	0.215
Myofascial pain syndrome	308,056.1	30,384.8	250,665.3	32,462.0	< 0.001*	0.589
Carpal tunnel syndrome	25,432.8	1847.9	24,661.1	1772.2	0.254	0.952
Tennis elbow	104,105.0	5741.3	105,039.4	6352.0	0.641	0.726
Frozen shoulder	125,905.2	5736.7	138,692.6	10,798.7	< 0.001*	0.998
Rheumatoid arthritis	77,551.2	3794.5	77,546.9	3534.7	0.776	0.720
Gout	106,692.5	7730.2	111,602.8	6946.7	0.043*	0.828
Hip fracture	15,288.6	787.5	15,051.6	587.7	0.365	0.227
Distal radius fracture	26,165.2	1908.6	24,960.8	2265.0	0.078	0.126
Spine fracture	42,182.2	2432.6	41,629.8	2613.4	0.407	0.113

Data are presented as mean and SD

*Mann–Whitney U test, significance at < 0.05

^†^Levene’s test in non-parametric data

COVID-19, coronavirus disease; SD, standard deviation

### Incidence of Orthopedic Diseases stratified by sex

In the subgroup analysis, both the male and female groups showed similar results as the first analysis: the incidence of myofascial pain syndrome decreased and that of frozen shoulder increased in both sexes (Table 2). However, the incidence of gout increased only in the male group (*P* = 0.036). Significant changes were found in only two diseases in the male group: the incidence of tennis elbow increased (*P* = 0.032), whereas that of distal radius fracture decreased (*P* = 0.007). In the female group, the incidences of lumbar disc disorders (*P* = 0.012) and carpal tunnel syndrome (*P* = 0.034) significantly decreased.

**Table 2:** Incidence of diseases before and during COVID-19, stratified by sex

Diseases	Before COVID-19		During COVID-19		*P*-values of difference	
	Mean	SD	Mean	SD	Mean	Variance^†^
**Men**						
Cervical disc disorder	71,397.4	3851.4	72,333.3	3843.9	0.660	0.257
Lumbar disc disorder	157,826.7	8903.7	156,721.7	8982.7	0.698	0.331
Forward head posture	44,473.9	3544.1	45,528.8	3925.2	0.422	0.177
Myofascial pain syndrome	123,731.5	13,708.3	100,217.7	12,873.1	< 0.001*	0.662
Carpal tunnel syndrome	5842.3	424.3	6119.9	489.6	0.052	0.099
Tennis elbow	52,048.4	2948.4	54,322.8	3392.6	0.032*	0.362
Frozen shoulder	50,493.9	2081.8	55,423.7	3805.6	< 0.001*	0.946
Rheumatoid arthritis	16,921.2	801.9	17,108.0	765.8	0.623	0.506
Gout	101,294.0	7234.0	106,038.2	6546.1	0.036*	0.946
Hip fracture	4167.0	203.7	4184.4	185.0	0.551	0.572
Distal radius fracture	8872.1	1185.8	7814.7	1046.4	0.007*	0.447
Spine fracture	10,202.6	576.5	10,492.9	572.3	0.066	0.447
**Women**						
Cervical disc disorder	86,070.6	4608.3	85,720.4	5542.2	0.698	0.111
Lumbar disc disorder	208,029.6	13,603.2	196,515.1	14,748.6	0.012*	0.380
Forward head posture	56,383.6	3839.0	56,337.2	4910.8	0.959	0.285
Myofascial pain syndrome	184,324.6	16,967.9	150,447.6	19,687.2	< 0.001*	0.605
Carpal tunnel syndrome	19,590.5	1483.8	18,541.2	1364.1	0.034*	0.879
Tennis elbow	52,056.6	2943.6	50,716.6	2998.2	0.120	0.710
Frozen shoulder	75,411.3	3823.6	83,268.9	7053.9	< 0.001*	0.852
Rheumatoid arthritis	60,630.0	3002.6	60,438.9	2782.1	0.534	0.939
Gout	5398.4	509.7	5564.6	418.9	0.277	0.876
Hip fracture	11,121.6	605.4	10,867.1	414.9	0.162	0.067
Distal radius fracture	17,293.1	1622.5	17,146.1	1679.8	0.736	0.959
Spine fracture	31,979.6	1971.5	31,136.8	2129.5	0.178	0.034^†^

Data are presented as mean and SD.

*Mann–Whitney U test, significance at < 0.05

^†^Levene’s test in non-parametric data, significance at < 0.05

COVID-19, coronavirus disease; SD, standard deviation

### Incidence of Orthopedic Diseases Stratified by Age

Regarding age, all age groups showed a lower incidence of myofascial pain syndrome during the pandemic (Table 3). Frozen shoulder showed increased incidence in individuals 20–59 and ≥ 60 years old, whereas gout showed increased incidence in individuals 20–59 years old. In the 20- to 59-year age group, the incidences of carpal tunnel syndrome (*P* = 0.007) and spine fracture (*P* < 0.001) decreased. The incidence of distal radius fracture decreased in individuals 0–19 and 20–59 years old but increased in individuals aged ≥ 60 years. The variance in frozen shoulder increased during the COVID-19 pandemic in those younger than 60 years; meanwhile, the variance in rheumatoid arthritis increased in all age groups.

**Table 3:** Incidence of diseases before and during COVID-19, stratified by age

Diseases	Before COVID-19		During COVID-19		*P*-values of difference	
	Mean	SD	Mean	SD	Mean	Variance
**Age: 0–19 years**						
Cervical disc disorder	687.1	100.5	763.6	108.4	0.037*	0.557
Lumbar disc disorder	3870.3	357.3	3497.5	504.9	0.020*	0.711
Forward head posture	8628.5	1194.3	8105.3	1535.4	0.265	0.790
Myofascial pain syndrome	16,154.1	3300.1	10,642.5	3188.6	< 0.001*	0.218
Carpal tunnel syndrome	88.6	18.1	98.6	16.3	0.092	0.920
Tennis elbow	436.3	69.2	391.1	84.9	0.117	0.270
Frozen shoulder	161.9	23.8	155.4	38.7	0.560	0.023^†^
Rheumatoid arthritis	199.3	22.2	396.1	324.0	0.351	< 0.001^†^
Gout	294.4	31.7	310.5	35.3	0.170	0.530
Hip fracture	82.3	11.6	70.1	12.6	0.002*	0.888
Distal radius fracture	5684.2	1703.6	4493.6	1451.7	0.032*	0.433
Spine fracture	211.8	35.3	189.0	43.0	0.092	0.605
**Age: 20–59 years**						
Cervical disc disorder	95,046.9	4871.0	92,167.3	5237.7	0.103	0.813
Lumbar disc disorder	189,402.7	9890.0	175,428.6	10,010.7	< 0.001*	0.172
Forward head posture	72,632.5	4998.8	73,640.0	5791.3	0.468	0.069
Myofascial pain syndrome	164,495.3	20,564.5	122,363.3	11,316.8	< 0.001*	0.062
Carpal tunnel syndrome	16,482.1	1237.0	15,406.1	1035.7	0.007*	0.314
Tennis elbow	81,313.7	4436.0	80,037.5	4389.8	0.300	0.847
Frozen shoulder	60,103.6	2538.1	67,081.3	4935.0	< 0.001*	0.751
Rheumatoid arthritis	36,722.7	1920.6	39,343.4	6354.9	0.959	0.014^†^
Gout	71,492.7	5436.0	75,560.3	4908.5	0.023*	0.946
Hip fracture	1498.2	67.0	1420.3	76.7	0.002*	0.855
Distal radius fracture	8003.5	881.8	7271.3	513.0	0.001*	0.518
Spine fracture	5190.6	294.6	4711.0	260.9	< 0.001*	0.134
**Age: 60 + years**						
Cervical disc disorder	62,007.9	4057.1	65,389.6	4584.4	0.020*	0.591
Lumbar disc disorder	173,209.6	12,991.9	174,865.4	13,826.8	0.534	0.409
Forward head posture	19,691.2	1307.3	20,228.4	1695.6	0.147	0.690
Myofascial pain syndrome	127,642.5	9343.6	117,864.7	19,517.6	0.002*	0.721
Carpal tunnel syndrome	8890.1	723.4	9182.7	776.3	0.233	0.478
Tennis elbow	22,510.9	1631.1	24,774.6	2049.5	0.002*	0.941
Frozen shoulder	65,847.3	3541.7	71,691.9	6064.1	0.001*	1.000
Rheumatoid arthritis	40,680.1	2268.1	37,853.6	6281.0	0.679	0.017^†^
Gout	34,982.2	2322.2	35,803.6	2068.0	0.244	0.887
Hip fracture	13,716.8	738.1	13,569.9	538.9	0.569	0.302
Distal radius fracture	12,545.5	1219.5	13,260.4	1553.6	0.043*	0.905
Spine fracture	36,826.4	2242.3	36,773.5	2377.3	1.000	0.331

Data are presented as mean and SD.

*Mann Whitney U test, significance at < 0.05

^†^Levene’s test in non-parametric data, significance at < 0.05

COVID-19, coronavirus disease; SD, standard deviation

## Discussion

This study was conducted to validate the hypothesis postulating that the incidence and variance of a wide range of orthopedic diseases may have changed during the COVID-19 era. We assessed the monthly incidence of 12 orthopedic diseases common in primary clinics and compared the incidence of diseases before and during the COVID-19 pandemic. Based on our results, the incidence of each disease showed a different pattern and was not consistent across subgroups by sex and age.

Using the terms “orthopedic,” “musculoskeletal,” “joint disease,” “joint pain,” “fracture,” and “COVID-19,” we searched PubMed and Embase and defined our search for English articles before March 2022. Most literature analyzed the incidence of traumatic orthopedic diseases, and one meta-analysis showed that the number of fractures decreased by 43% during the COVID-19 pandemic (odds ratio [OR], 0.32; 95% confidence interval [CI], 0.16–0.66; *P* = 0.002; I^2^ = 89%, *P* < 0.001) [9]. We could not find any studies on the frequency and variance in common orthopedic diseases, leading us to conduct this study. The Korean government did not mandate a strict lockdown and used non-pharmaceutical interventions (NPIs), including suggesting voluntary work from home conditions and local quarantine around confirmed patients [16]. Although the Korean government used a limited social distancing program, individuals’ behaviors changed. In the early stage of the pandemic, 92.3% of survey respondents avoided using healthcare facilities [5]. However, the response to the warning signal slowed and weakened with the implementation of a stronger NPI for each surge [17]. Therefore, evaluating the variance of incidence was necessary.

Medical visits due to cervical disc disorder increased over time, whereas those for lumbar disc disorder decreased. However, these changes were not statistically significant in the present study. A few studies reported the impact of COVID-19 on musculoskeletal diseases in Korea. In one study, the number of visits for spinal diseases was compared with that during the previous 3 years, and both daily outpatient and emergency room visits significantly decreased [18]. In a previous study, the number of visits decreased at both a tertiary medical center and a private spine hospital. A survey of orthopedic surgery residents in Korea reported that working time significantly decreased (*P* < 0.001) during the pandemic [19]. The working time in operating rooms significantly decreased, and the authors presumed the reason for the cancellation of elective outpatient visits or surgery was due to the pandemic restrictions. The main risk factor for cervical and lumbar disc diseases is known to involve posture and heavy loads, respectively [20]. With this perspective, restrictions during the pandemic period may increase duration of indoor life and contribute to poor cervical posture. Furthermore, less physical activity and movement may reduce the factors that cause lumbar pain. The subgroup analysis in our study showed increased cervical disc disorders in those aged 0–19 years and decreased lumbar disc disorders in those aged 0–59 years. Further studies on the detailed mechanism of this change are needed.

The incidence of myofascial pain syndrome was significantly lower during the pandemic in our study, and the results were consistent by sex and age group. Although no study on myofascial pain syndrome after the pandemic exists, we could refer to studies on fibromyalgia, as they share a similar disease entity. In a survey conducted in Saudi Arabia, the prevalence of fibromyalgia in health workers was high (12.6–19.8%) during the pandemic [21]. However, respondents were mostly in their 40s, and the study only evaluated the early stage of the pandemic. Cavalli et al. described the dual effects of lockdown on patients with fibromyalgia [22]. Reduced physical activity and anxiety were the reasons for symptom worsening. However, regular physical activity and efficient work from home led to symptom improvement. A previous study on French patients with fibromyalgia reported similar results [23]. Since social demand was reduced by lockdown, patients had fewer constraints on the pace of life and could control their lives. Another common comorbidity with fibromyalgia is depression [24]. Previous studies dealing with subjects of different natures in the early days of the pandemic can be referenced. In a community survey of 217,333 Korean participants, the rate of depression was lower during the pandemic (OR, 0.95; 95% CI, 0.91–0.98; *P* = 0.004) than it was before the pandemic [25]. However, caution is needed, as the actual number of patients experiencing these conditions may be higher than the number of visits to the hospital.

We found a significantly increased incidence of frozen shoulder during the pandemic in this study. In a study of an Irish shoulder clinic, the incidence of frozen shoulder significantly increased to 39.8% (*P* < 0.001) during the pandemic [26], and the waiting time for an appointment was similar before and after the pandemic. Furthermore, Demyttenaere et al. also suggested that poor glycemic control of patients with diabetes mellitus could have influenced the results. During the pandemic, changes in the control of diabetes were also observed. The mean hemoglobin A1C level was higher (male, *P* < 0.01; female, *P* = 0.41) than that of the non-pandemic cohorts in Korea [27]. A previous study suggested that deleterious effects of social distancing were more marked in socially active patients with diabetes mellitus. Although still controversial, diabetes is believed to be a poor prognostic factor for frozen shoulder [28]. Other factors that affect shoulder pain can also be considered. The vaccine was introduced late in Korea (early 2021), and it could also have caused shoulder pain. Among patients with shoulder injury related to vaccine administration, patients without prior history of shoulder pain, limited shoulder range of motion, and subacromial-subdeltoid bursitis were the most common demographics [29]. As frozen shoulder is affected by a variety of predisposing factors, further studies are needed.

In the present study, two types of inflammatory arthritis were evaluated: gout and rheumatoid arthritis. The incidence of gout increased with statistical significance. In a study of a Mexican gout clinic, there were nine times more flares (*P* = 0.01) and higher urate levels (*P* = 0.016) during the pandemic than in the pre-pandemic period [30]. Gout flares were also common during the pandemic in a survey study in the United States: 38% of respondents had multiple flares per month [31]. In a previous study, one-third of the participants reported moderate or severe psychological distress. The frequency of visits by patients with gout may have increased because flares and stress were more common in the pandemic period. Changes in eating habits and lifestyles during the pandemic could have affected uric acid levels. However, there are no Korean data on eating habits during the pandemic; thus, foreign papers can be referenced. In a survey study in Italy, 34.4% of respondents had increased appetite, and 48.6% of them had the perception of weight gain during lockdown [32]. An Italian cross-sectional study reported a 23.5–42.5% increase in consumption of sweet food or salty snacks, and approximately half of the respondents consumed more food during lockdown [33]. A review article also presented deleterious effects of lockdown, including increased intake of processed meat and reduced intake of fruits and vegetables [34]. These factors may have increased the incidence of gout. When referring to studies from other countries, it can be assumed that Korea will show similar results.

The incidences of hip, distal radius, and spine fractures during the pandemic did not differ significantly compared with those of the pre-pandemic period in this study. A meta-analysis reported that the number of fractures presented to hospitals declined by 43% (35–50%) during the pandemic [9]. All studies included in this previous meta-analysis were published in countries that mandated lockdown, and the study period was short: 1–2 months from the beginning of 2020. As mentioned earlier, the Korean government used NPIs without mandating strict lockdown [16], and this policy may have led to different results [35]. In a study in a spine clinic of a Korean tertiary hospital, the average number of both elective and emergency surgeries during the pandemic period was similar to that of the pre-pandemic period [18]. Ham et al. suggested that patients requiring surgical intervention still visited the hospital regardless of the pandemic situation. Another point to consider is the changing trend of incidence. Referring to studies done early in the pandemic, after social distancing in Korea, fewer injuries were registered in the National Emergency Department Information System database [36]. However, this effect gradually decreased over time; the estimate of step-change for injury incidence rate per 100,000 person-days was −3.23 (95% CI, −43.4 to −2.12). The authors suggested psychological fatigue with social distancing as a reason. In our paper, the incidence of the three fractures decreased until March 2020 and then increased gradually, showing a similar incidence as that before the pandemic. Since the pandemic persisted and NPIs were repeated, the effect of the NPIs reduced, the duration of the effect became shorter, and the intensity decreased less than a year after the onset of the pandemic owing to people’s exhaustion [17].

This study is the first to demonstrate the variance in common orthopedic diseases during the COVID-19 pandemic period and to compare the incidence with that before pandemic. However, this study has several limitations. First, diseases were identified by ICD-10 codes in the claims database. Therefore, coding/mismatching/misclassification errors are possible, and the primary diagnosis and other diagnoses cannot be distinguished. Second, we analyzed health claims data using diagnostic codes and thus failed to consider subclinical status, undiagnosed diseases, or overdiagnosis cases. Third, the ability to determine definite causality was limited because our study had an observational design. Our study could not confirm the pathophysiological mechanism of changes in the incidence and variance, as only means and standard variations were calculated. Fourth, several variables related to musculoskeletal disorders could not be analyzed, including underlying gait abnormalities, weakness or palsy, or deformity. We did not consider other possible confounding factors, such as obesity, body mass index, smoking, alcohol drinking, and past medical histories. Furthermore, physical inactivity, social isolation, subjective health status, and education level may have influenced the medical usage of participants. However, this study used national health insurance system data, which covers the entire population without an exception. By including a large population without missing participants, statistical power was obtained [25]. Further longitudinal studies adjusting for potential confounding factors are needed to confirm our findings. Fifth, we analyzed monthly incidence in the 2 years preceding the pandemic period, which can be short-term data. However, we believe that using the most recent data would be appropriate for comparison, and the distribution of disease incidence could be observed. Sixth, the heterogeneity in diseases may exist, and each treatment method may be different. Further research is needed not only on the diagnostic code but also on the treatment method implemented. Lastly, since there have been differences between countries’ policies, the results of this paper might not be generalizable to other countries. The United States and Europe implemented lockdowns, and outpatient appointments and elective surgeries were canceled or reduced during this period [10]. Such differences in government policy may have influenced the medical usage of patients. Furthermore, varying levels of social distancing could have affected the incidence of diseases. A future study that reflects the movement of people and the intensity of outdoor activities according to policy changes is needed.

## Conclusions

The incidence of orthopedic diseases varied during the COVID-19 pandemic period in the Korean population. Myofascial pain syndrome had a lower incidence during the pandemic than during the pre-pandemic period, whereas frozen shoulder and gout showed a higher incidence during the pandemic than during the pre-pandemic period. In most cases, the incidence decreased at the beginning of the pandemic and then increased thereafter. Despite the government’s social distancing program, diseases showed an increasing pattern after 1 year of pandemic. With this study, physicians can perceive the effect of social isolation on the incidence and variance of different diseases and recognize the need to redistribute available resources and restructure the healthcare system.

## Electronic supplementary material

Below is the link to the electronic supplementary material.

Supplementary Material 1

Supplementary Material 2

Supplementary Material 3

Supplementary Material 4

## Data Availability

The data that support the findings of this study are available from the Korean NHIS (http://nhiss.nhis.or.kr/), but restrictions apply to the availability of these data, which were used under license for the current study and thus are not publicly available. Data are, however, available from the corresponding author upon reasonable request and with permission of the Korean NHIS.

## Abbreviations

CI: Confidence interval
COVID-19: Coronavirus disease
ICD-10: International Classification of Diseases, Tenth Revision
NHIS: National Health Insurance Service
NPI: Non-pharmaceutical intervention
OR: Odds ratio
