# Supplementary figures and images for: Changes in the mean incidence and variance of orthopedic diseases before and during the COVID-19 pandemic in Korea: a retrospective study
Source: BMC Musculoskelet Disord. 2023 Jul 1;24:540. doi: 10.1186/s12891-023-06634-0 (PMC10314473; doi:10.1186/s12891-023-06634-0)

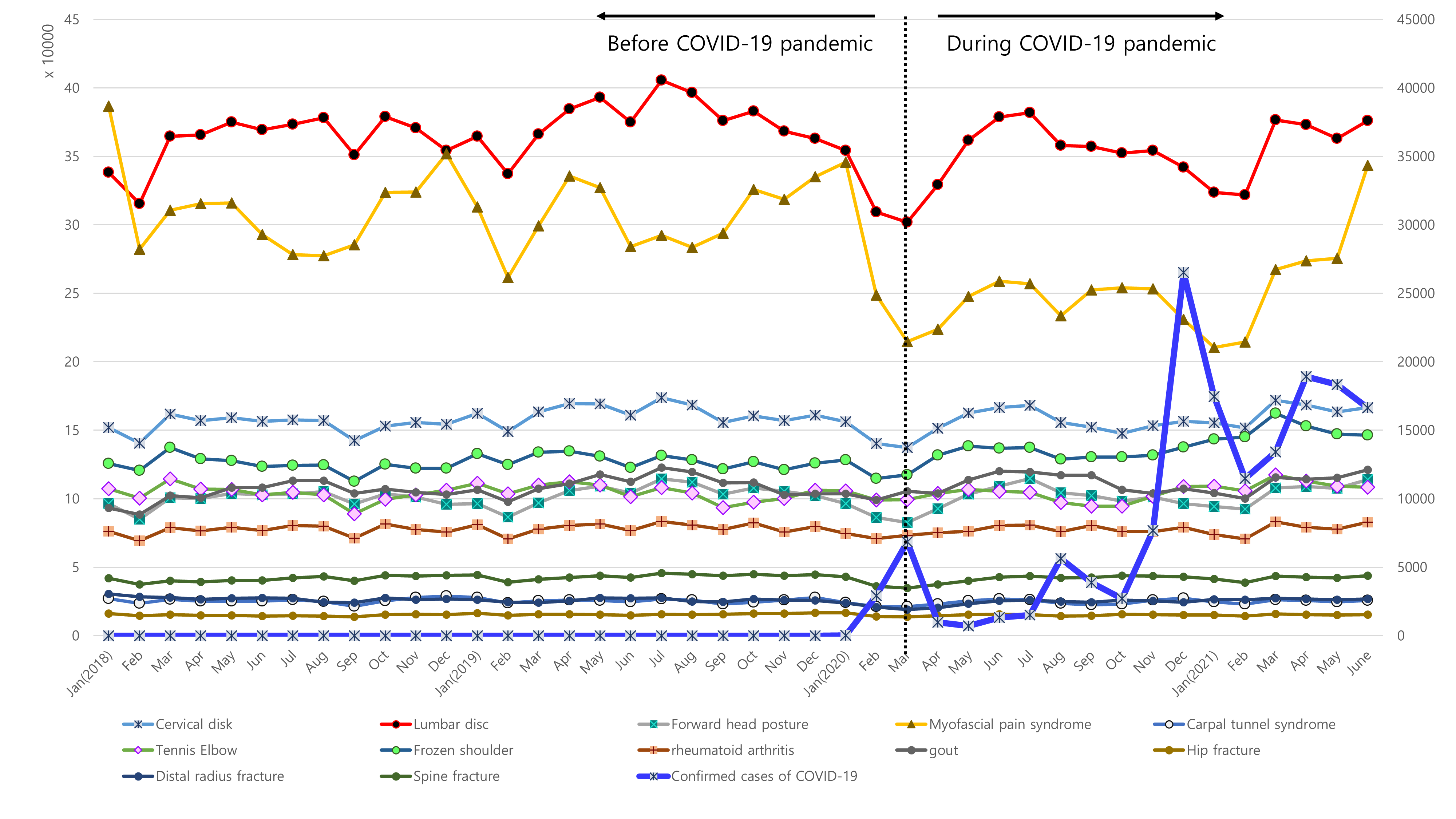

Supplement: Supplementary file 1 — Supplementary Material 1 [file 12891_2023_6634_MOESM1_ESM.tif]

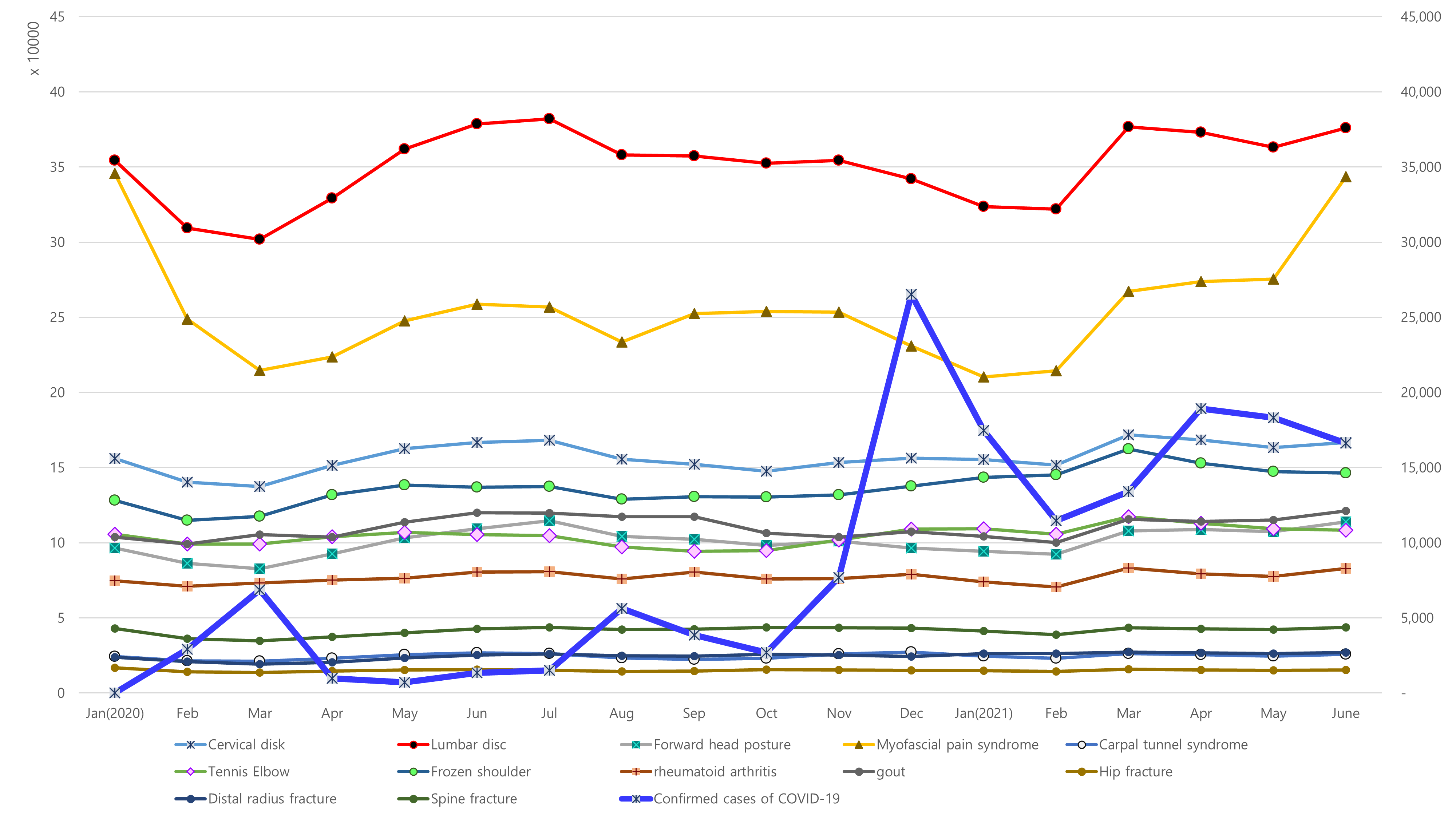

Supplement: Supplementary file 2 — Supplementary Material 2 [file 12891_2023_6634_MOESM2_ESM.tif]

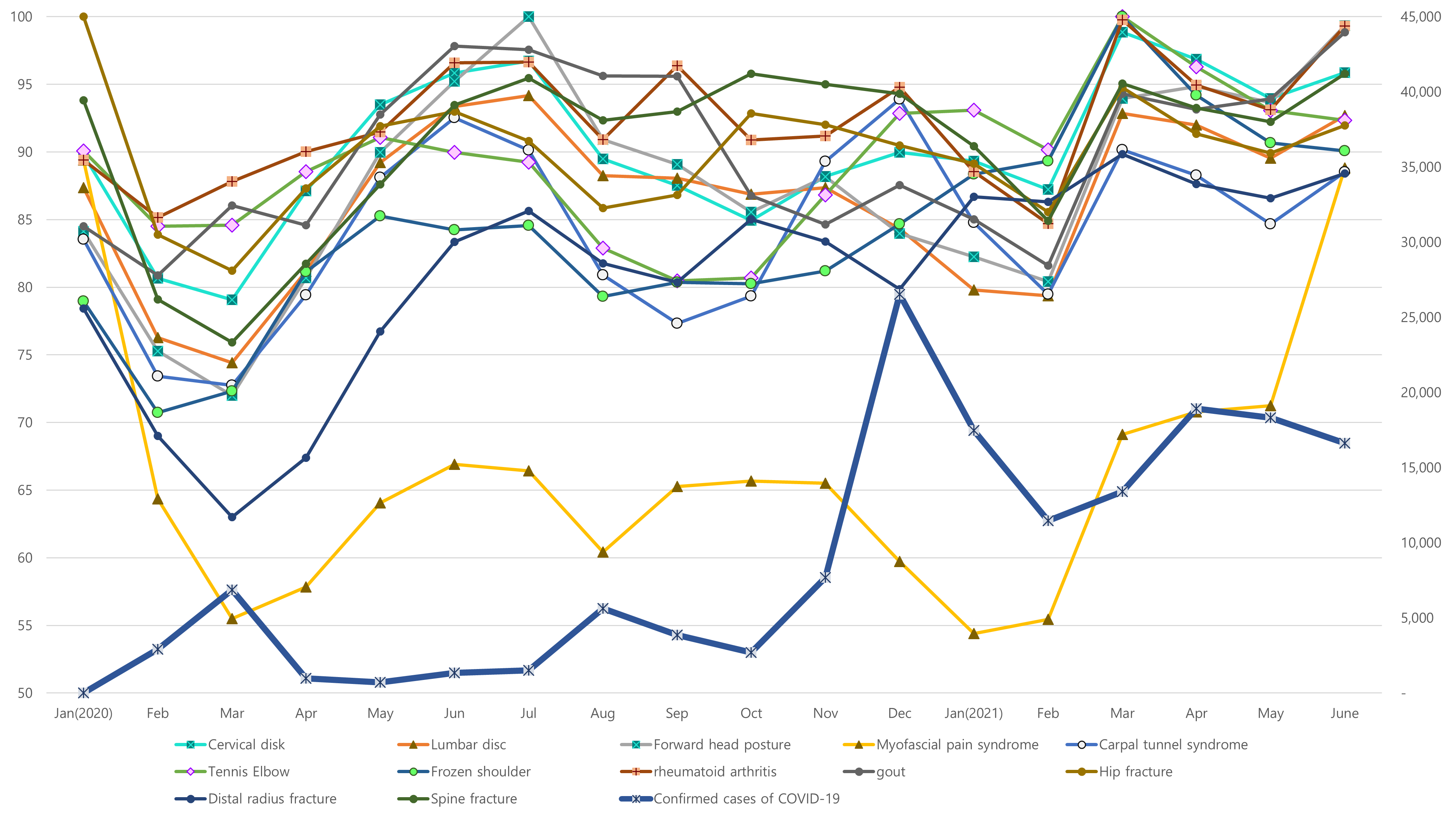

Supplement: Supplementary file 3 — Supplementary Material 3 [file 12891_2023_6634_MOESM3_ESM.tif]
