# Supplementary material for: Changes in the mean incidence and variance of orthopedic diseases before and during the COVID-19 pandemic in Korea: a retrospective study
Source: BMC Musculoskelet Disord. 2023 Jul 1;24:540. doi: 10.1186/s12891-023-06634-0 (PMC10314473; doi:10.1186/s12891-023-06634-0)
Supplement: Supplementary file 4 — Supplementary Material 4 [file 12891_2023_6634_MOESM4_ESM.docx]

**Additional file 1**: The monthly incidence of orthopedic diseases and cases of COVID-19 during 2018, 2019, 2020, and 2021. TIFF, This graph shows the monthly incidence of common orthopedic diseases and monthly cases of COVID-19 during 2018, 2019, 2020, and 2021. The study duration was divided into two periods: “before COVID-19” (until February 2020) and “during COVID-19” (from March 2020).

**Additional file 2**: The monthly incidence of orthopedic diseases and cases of COVID-19 during pandemic. TIFF, This graph shows the monthly incidence of common orthopedic diseases and monthly cases of COVID-19 after the first patients with COVID-19 were identified in Korea (January 2020).

**Additional file 3**: The relative incidence of orthopedic diseases and COVID-19 during pandemic. TIFF, This graph shows the relative incidence (%) of orthopedic diseases compared to each maximum monthly incidence and the monthly confirmed cases of COVID-19 over the study duration.
